# Can Disruption of Circadian Rhythms Be Linked to Radiation-Induced Acute Myeloid Leukaemia?

**DOI:** 10.3390/cancers18142255

**Published:** 2026-07-14

**Authors:** Aleksandra Czyzak, Gráinne O’Brien, Milagrosa Lopez-Riego, Lourdes Cruz-Garcia, Christophe Badie

**Affiliations:** 1 Radiation Effects Department, RCCE, UK Health Security Agency, Chilton, Didcot, Oxfordshire OX11 0RQ, UKmilagrosa.lopezriego@ukhsa.gov.uk (M.L.-R.);; 2 Department of Oncology, University of Oxford, Oxford OX4 1ET, UK

**Keywords:** acute myeloid leukaemia, clock genes, circadian rhythms disruption, ionising radiation, DNA damage response, shift work, ATM, p53, PER1/2/3

## Abstract

Acute myeloid leukaemia (AML) remains a highly lethal malignancy with poor prognosis in adults above 60 years old and often occurs after radiation exposure. Emerging evidence suggests that circadian rhythm disruption, prevalent in shift workers, may contribute to cancer development. Clock genes (CGs) regulate fundamental cellular processes, including the DNA damage response (DDR), cell cycle progression, and haematopoiesis. In the absence of substantial experimental data, this review examines the potential pathways linking circadian clock dysregulation to radiation-induced AML (rAML) and evaluates how temporal disruption may modulate leukaemogenesis and radiation-induced effects. The evidence was synthesised on core clock components (*BMAL1*, *CLOCK*, *PER*, *CRY*, *REV-ERB*, *ROR*), their dysregulation in AML, and their roles in radiation response. Epigenetic and post-transcriptional regulatory mechanisms, including m^6^A RNA modification and sirtuin-mediated chromatin remodelling, were evaluated for their contribution to circadian-regulated DNA repair capacity. Multiple CGs demonstrated aberrant expression in AML, with *BMAL1* showing tissue-specific dysregulation, and *PER1/2/3* was consistently downregulated in peripheral blood. Clock proteins directly regulate DNA damage checkpoints through interactions with ATM/CHK2 and p53 pathways. Circadian disruption enhances inflammatory signalling, promotes accumulation of myeloid-derived suppressor cells, and accelerates immune senescence. Moreover, radiation exposure modulates CG expression, which may alter repair fidelity and increase leukaemogenic risk. Understanding these connections in the context of disrupted circadian rhythms could help identify at-risk populations and improve shift workplace health policies.

## Simple Summary

Acute myeloid leukaemia (AML) is a rapidly progressing blood cancer with poor survival rates, particularly in adults over 60 years old. Exposure to ionising radiation (IR) can contribute to AML development, while modern shift work patterns that disrupt the body’s natural daily rhythms may increase cancer risk. Clock genes (CGs) help regulate critical processes, including DNA repair and blood cell production. When these rhythms are disrupted, through nightshift work or irregular sleep, the body’s ability to repair damaged DNA may be compromised. However, direct experimental evidence linking circadian disruption combined with IR exposure to AML development is currently limited. This review examines how radiation exposure, in the context of disrupted circadian rhythm, might lead to AML development. Understanding these connections could help identify at-risk populations and potentially lead to better treatment timing strategies and improved shift workplace health policies.

## 1. Introduction

Acute myeloid leukaemia (AML) is a rapidly progressing haematological group of malignancies characterised by the clonal expansion of immature haemopoietic stem cells (HSCs) in the bone marrow [1]. Clonal expansion occurs at the expense of normal daughter cell production, such as red blood cells, platelets, and white blood cells. The onset of AML symptoms typically occurs within a few weeks. It is characterised by nonspecific symptoms, such as fatigue and shortness of breath, recurrent infections, and a tendency to bruise and bleed due to bone marrow failure [2].

In total, 150,000 new cases of AML are diagnosed worldwide each year, making it the most common form of acute leukaemia in adults [3]. Despite advances in traditional therapies (radiotherapy, chemotherapy, bone marrow transplantation, or targeted therapies), the prognosis remains poor, with overall five-year survival rates for those over 60 years old receiving intensive treatment at 20%, and only 2% of the population surviving a year without any treatment [4]. The multifactorial nature of AML pathogenesis involves acquired somatic mutations and environmental exposures such as ionising radiation (IR) [5]. IR can act as a cancer initiator, promoter, or accelerator, depending on various factors, including the latency period to diagnosis, radiation quality, dose, dose rate, route of exposure, and the sensitivity, kinetics, and organisational structure of cells within the target tissue or organ. Additional factors, such as sex, age at exposure, and hormonal levels, may also influence the risk of developing radiation-induced AML (rAML) [6]. rAML demonstrates the leukaemogenic potential of IR. Historically, leukaemic cases were reported following atomic bomb exposures and nuclear accidents, with rAML being the most commonly occurring (Hiroshima, Nagasaki or Chornobyl) [7,8]. Nowadays, rAML occurs more frequently as secondary malignancies in patients treated with radiotherapy, often in combination with chemotherapy and known as therapy-related AML, for a range of cancers such as breast, prostate, genitourinary, head and neck, lymphoma or myeloma, the mechanism of which remains unidentified [9]. Some evidence suggests that the synergistic effects of radiotherapy and chemotherapy in these patients, by damaging DNA repair mechanisms, together with potential pre-existing mutations in hematopoietic stem cells and a radiation-induced inflammatory bone marrow microenvironment, may contribute to the accelerated progression of mutated or premalignant clones [10,11].

Modern lifestyle patterns associated with industrialised societies have introduced circadian rhythm disruption as a relevant oncogenic factor [12]. Chronic misalignment between endogenous circadian clocks and environmental or social timing, as occurs in night-shift workers, individuals exposed to artificial light at night, or those with irregular sleep schedules, disrupts this temporal organisation. Shift work is prevalent in the modern world, with 19% of people in Europe working night shifts, as reported by the Sixth European Working Conditions Survey, and so the long-term health implications of these individuals are of particular concern [13]. In 2007, the International Agency for Research on Cancer (IARC) listed shift work that involves circadian disruption as a probable human carcinogen, based on sufficient evidence from animal studies and limited evidence from human epidemiological research. The latter reports an increased risk for certain cancer types such as breast, prostate, and colorectal cancers [14].

There are few studies investigating the link between shift work and haematological cancers. A case–control study in Montreal detected a greater than 2-fold increase in risk of non-Hodgkin’s lymphoma (NHL) in men who worked night shifts. Conversely, a study on female nurses from the Nurses’ Health Study (NHS)-I cohort by Gu et al. reported no evidence of increased mortality from NHL for those working rotating night shifts [15]. A study on male German chemical workers also reported no increased risk of NHL but an increased risk of leukaemia in shift workers in comparison to day workers [16]. Another cancer mortality study on male chemical manufacturing plant workers in New York State found an increased risk of NHL [17]. The multi-case–control study (MCC) in Spain investigating an association between night-shift work and Chronic Lymphocytic Leukaemia (CLL) found that overall, there was no increased risk of CLL. However, a long-term night shift was positively associated with CLL for those with rotating rather than permanent night shifts [18]. A large case–control study on night-shift workers in Nordic countries found no association with haematological cancers but a small non-significant increased risk for leukaemia, which was significant for AML in men [18]. The Working Group determined that for haematopoietic cancers, no conclusions can yet be made for an increased risk with night-shift work due to the small number of studies reporting results and their inconsistent findings [19]. The inconsistency in reported cancer risk could be due to issues such as a lack of clarity in the definition of night-shift work, different populations studied, and different cancer outcomes, such as incidence or mortality. To assess the cancer risk in low-incidence cancer such as AML, larger-cohort studies are needed. Many occupational cohorts lack enough AML cases for subtype-specific analysis, which has consequently limited epidemiological studies on increased risk of leukaemia. However, since the IARC report in 2020, a large-scale study involving over 70,000 US female nurses has now reported an increased risk of several types of hematopoietic cancers, including myeloid leukaemias, again in those with more than 15 years of rotating night-shift work [20].

The classification of night-shift work as ‘probably carcinogenic to humans’ is primarily based on experimental evidence from animal models providing valuable mechanistic insight [19]. Alterations of the circadian clock have been linked to cancer development, and core circadian clock transcription factors have been shown to act as tumour-suppressors. *mPer2m/m* mice develop malignant lymphomas after 16 months after IR exposure at 71% incidence, while only 5% of irradiated wild-type mice develop lymphomas [21]. A role for clock genes in AML development has also been established with the disruption of *Clock* and *Bmal1* by shRNA knockdown, targeted mutation, or deletion impairing murine AML proliferation *in vivo* and human cell lines *in vitro* [22]. Moreover, population-based case–control studies have shown that polymorphisms in the clock genes *CRY2* and *NPAS2* and alterations in their expression are associated with an increased risk of NHL [23]. Altogether, these studies point towards a complex relationship between circadian disruption and leukaemic development.

It is suggested that night-shift schedules can desynchronise and blunt peripheral clock gene (CG) expression, while appropriately timed bright light can acutely realign these molecular rhythms [24]. Emerging evidence points to a direct role of CG dysregulation in haematological malignancies [25,26]. Studies have shown that components of the core clock, including BMAL1, CLOCK, PER, and CRY gene families, intersect with cell cycle checkpoints, DNA damage responses, and apoptotic signalling [13]. Perturbations in these genes alter hematopoietic stem cell (HSC) homeostasis and promote malignant transformation [27]. A study on healthy adults exposed to a 4-day simulated night shift (10 h sleep delay) with a dense 24 h time-series sampling of peripheral blood mononuclear cells (PBMCs) for circadian transcriptome profiling found loss of circadian coordination and disruption of NK cell-mediated immunity as well as *JUN/AP-1* and *STAT* signalling, suggesting plausible immune-stress mechanisms that may link night-shift work to adverse health effects [28]. Desynchrony was also shown in central markers (melatonin and cortisol) and distinct PBMC expression; however, *PER1* rhythms in PBMCs appeared similarly phased, suggesting downstream transcriptomic reorganisation rather than a uniform shift in core clock genes [29]. Importantly, another study on PBMCs from simulated night-shift workers revealed altered rhythmicity of cancer hallmark genes and pathways (including key DNA repair genes) together with increased endogenous and IR-induced DNA damage following circadian dysregulation compared to non-shift controls [30]. To our knowledge, similar studies of induced circadian dysregulation in animal models for the evaluation of long-term effects, have predominantly focused on non-leukaemic endpoints [31,32] but lymphomas/leukaemias presented at a high incidence in rats under abnormal lighting schemes [33]. However, *in vivo* work directly linking circadian disruption through simulated shift work schemes to AML, and in combination with IR exposure in particular, is currently missing.

This review examines the mechanistic pathways through which circadian clocks regulate DNA repair response, haematopoiesis, and leukaemogenesis. It synthesises evidence linking circadian disruption with radiation-induced damage, highlights alterations of specific CGs in AML, and discusses emerging translational opportunities in chronoradiotherapy and occupational health. AML is the most common occurring type of leukaemia after radiation exposure and so for this reason, we will focus solely on AML for the remainder of the review, albeit the observations gathered herein may potentially apply to a broader group of leukaemia types. The goal is to outline how circadian biology can inform both cancer prevention and therapeutic optimisation in AML after radiation exposure, shaping future diagnostic, regulatory, and treatment pathways.

## 2. Acute Myeloid Leukaemia and Radiation

The high heterogeneity of AML has been recognised since the early analysis of AML karyotypes [34]. Currently, the World Health Organisation identifies 25 subtypes of AML [35]. The mutational landscape of AML frequently includes recurrent alterations in genes regulating transcription (*RUNX1*, *CEBPA*), signalling (*FLT3*, *KIT*), and epigenetic machinery (*DNMT3A*, *TET2*, *IDH1/2*), highlighting the disease’s heterogeneous origins. While these somatic events underpin leukaemogenesis, environmental and therapeutic exposures, particularly IR, serve as extrinsic contributors [36]. 

The leukaemogenicity potential of IR depends on multiple parameters, including dose, dose rate, radiation quality, and fractionation schedule [37]. A persistent challenge in understanding rAML is defining the initiating cellular and molecular events. DNA lesions, such as point mutations, small insertions or deletions, and translocations, are primary drivers of cancer. However, the presence of such mutations does not invariably result in malignancy, indicating that additional promoting factors are required for clonal outgrowth [38]. This “multi-hit” model suggests that IR is one of several cooperating events that contribute to complete leukaemic transformation [39]. Disruption of circadian rhythms may serve as one such cofactor; therefore, it is essential to investigate how dysregulation of circadian rhythms may modify AML risk.

## 3. Clock Genes, DNA Integrity, and Cell Cycle Regulation

Circadian clocks are molecular timekeeping systems that synchronise physiological processes with environmental cycles [40]. At the cellular level, circadian rhythms are generated through transcriptional–translational feedback loops (TTFLs) composed of core CGs and proteins, including *CLOCK*, *BMAL1*, *PER*, *CRY*, *REV-ERBs*, *RORs*, and *CK1ε* [41]. In the primary loop, heterodimers of CLOCK and BMAL1 bind to E-box promoter elements to activate transcription of *PER* and *CRY* genes. The translated proteins accumulate, are phosphorylated by CK1ε, and re-enter the nucleus to inhibit CLOCK–BMAL1 activity, thereby closing the negative feedback loop. Auxiliary loops, mediated by REV-ERBα/β and RORα/β/γ, regulate *BMAL1* transcription and stabilise oscillations. Additional repressors such as DEC proteins and co-factors, including NPAS2, NCORs, and PPARs, introduce further complexity [42]. These clock-controlled networks extend beyond rhythmic gene expression to intersect with the cell cycle and DNA integrity checkpoints.

**Figure 1:**
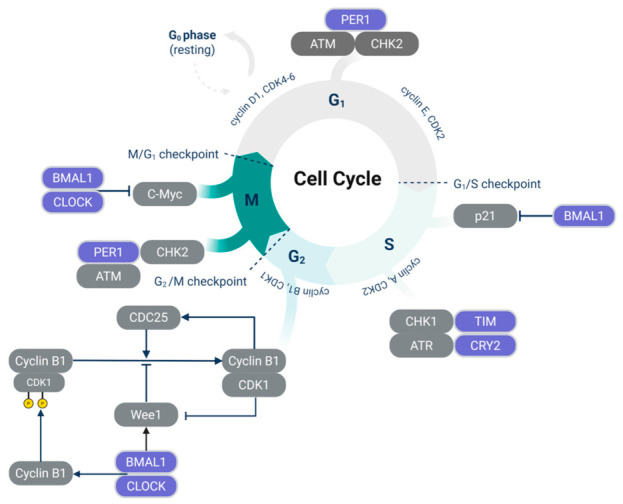
Circadian clock regulation of the cell cycle and DNA damage response. Mammalian cell cycle phases (G_1_, S, G_2_, M) and key checkpoint controls, highlighting interactions with circadian clock components. Core clock proteins BMAL1 and CLOCK influence cell cycle progression via C-Myc and p21 regulation at the G_1_/S and M/G_1_ checkpoints. PER1 and ATM cooperate with CHK2 to regulate the G_2_/M checkpoint. TIM and CRY2 modulate the S phase checkpoint via ATR and CHK1 signalling. Cyclin B1/CDK1 and its regulation by CDC25 and Wee1, with modulation by BMAL1 and CLOCK. Modified from Farshadi, van der Horst, and Chavex (2020) [43]. Created in BioRender. O’Brien, G. (2026) https://BioRender.com/ark4bxa accessed on 18 May 2026.

Studies in mammalian tissues reveal temporal gating of cell cycle transitions, DNA synthesis, and mitosis, with clock proteins directly regulating checkpoint regulators. For instance, CLOCK–BMAL1 complexes drive *c-MYC* transcription during the G0/G1 transition, coupling growth signalling to circadian time. Conversely, BMAL1 suppresses p21, a cyclin-dependent kinase inhibitor; loss of BMAL1 leads to elevated p21 and altered G1/S progression. At the G2/M checkpoint, BMAL1 and CLOCK regulate the expression of Wee1 kinase, which inhibits CDK1 and delays mitotic entry [43]. Knockdown of *CLOCK* or *BMAL1* reduces Cyclin B1 levels, impairing checkpoint fidelity [44]. These findings demonstrate that circadian clocks do not merely track time but actively gate cell proliferation (Figure 1).

A central mechanism linking circadian clocks to genome maintenance is the DNA damage response (DDR). PER1 has been shown to associate with the ATM–CHK2 complex, a sensor-effector pathway activated by IR–induced double-strand breaks. Through this interaction, PER1 enhances checkpoint activation, promoting either DNA repair or apoptosis. NPAS2 has been implicated in maintaining genomic stability by regulating DDR-related gene expression, while CRY proteins influence ATR/CHK1-mediated responses to replication stress [45]. Together, these interactions indicate that circadian clocks establish a temporal architecture of DNA repair capacity, modulating cellular outcomes following genotoxic stress.

The oncological implications of circadian–cell cycle crosstalk are supported by evidence from cancer models. Screens in mice reveal the heterodimeric circadian rhythm TFs *Clock* and *Bmal1* as genes required for the growth of AML cells *in vitro* and *in vivo* [22].

## 4. Circadian Control of Haematopoiesis

Haematopoiesis is a tightly regulated process that ensures the continuous renewal of blood and immune cells from multipotent HSCs [46]. This process follows circadian oscillations that synchronise cell proliferation, differentiation, and mobilisation with the body’s systemic physiological needs [47]. Early research showed rhythmic variations in bone marrow engraftment and mitotic activity, indicating the presence of intrinsic oscillators within the haematopoietic niche. Later studies confirmed that circadian rhythms regulate HSC trafficking, with peak egress into the bloodstream occurring during the organism’s rest phase in both rodents and humans [26]. CGs such as *Per1*, *Per2*, *Bmal1*, and *Clock* show circadian expression patterns in murine bone marrow, and approximately 6–9% of the human blood transcriptome fluctuates rhythmically across the day–night cycle [48]. Circadian control extends beyond HSC trafficking to influence lineage commitment and immune cell function [49]. For example, rhythmic regulation of cell cycle genes coordinates progenitor proliferation, while oscillations in cytokine production and receptor expression modulate immune readiness. These rhythms ensure that haematopoietic and immune responses are optimally timed to predictable environmental stressors such as pathogen exposure during the active phase [50]. Disruption of circadian clocks, whether by genetic manipulation or environmental perturbation, disturbs these dynamics and impairs immune competence [51]. Studies of CGs knockouts underscore the functional relevance of circadian regulation in haematopoiesis. Mice deficient in *Bmal1* exhibit premature ageing phenotypes, shortened lifespan, and impaired HSC self-renewal, reflecting the role of circadian oscillators in sustaining stem cell homeostasis [42].

Disruption of this circadian regulation of haematopoiesis can also affect progression of clonal hematopoiesis (CH). CH is the accumulation of somatic mutations in bone marrow HSCs that activates inflammation, gives these cells a growth advantage and can lead to the development of AML. A study on the effects of sleep fragmentation in atherogenic mice with CH mutations *Jak2V^617F^* mutations or *Tet2* loss of function reported blood monocyte and neutrophil and bone marrow myeloid progenitor expansion. Sleep fragmentation also produced an activation of the inflammatory genes *Il1b*, *Il6*, *Nlrp3*, *Aim2*, *Gsdmd*, *Casp1*, and *Il18*, which was reported in *Jak2V^617F^* macrophages [52]. Circadian disruption also exerts epigenetic effects by accelerating the epigenetic age of female blood leukocytes [53] and altering the human epigenome, promoting myeloid differentiation [54].

Circadian regulation of haematopoiesis provides critical insight into radiation sensitivity. HSCs cycling in and out of quiescence have similar radiosensitivity but are thought to use different DNA repair mechanisms. If circadian rhythms are altered, this serves as another point to be explored in tumorigenesis [55].

## 5. Clock Gene Dysregulation in AML and Radiation Response

**Figure 2:**
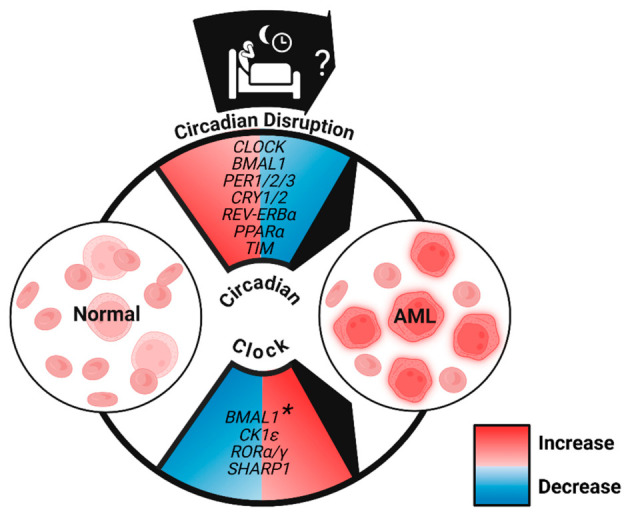
Circadian clock gene transcriptional dysregulation in the peripheral blood (* or bone marrow) of AML patients. Colours indicate direction of reported change in CG expression (red increase; blue decrease) in AML relative to normal conditions, represented by the arrow. Studies have shown that *CLOCK* and *BMAL1* are downregulated or silenced in leukaemic cells, with a 5-fold decrease in *BMAL1* when compared with healthy individuals. *PER1/2/3* and *CRY1/2* are downregulated in human studies and upregulated in AML remission. Increased blast cells in bone marrow correlated with decreased expression of *PER1* and *PER3*. *TIM* is downregulated in AML and upregulated in remission. Interestingly, *BMAL1* expression was found to be significantly upregulated in bone marrow samples * from human AML patients in comparison to healthy donors. Upregulated *CK1*s promote *PER* turnover, facilitating cyclin accumulation and cell cycle progression. *RORα* has been overexpressed in AML cells, and *RORγ* was upregulated in a study of volunteers with AML, as an essential factor for the Th17 cell lineage. *SHARP1* is upregulated in MLL-AF6 AML. All observations derived from peripheral blood, with the exception of BMAL1* expression in bone marrow (as indicated by *) Created in BioRender. Lopez Riego, M. (2026) https://BioRender.com/zsdjus9 accessed on 18 May 2026.

Dysregulation of CGs has been documented across multiple malignancies, including haematological cancers [56]. In AML, these genes represent both mechanistic drivers of leukaemogenesis and potential biomarkers for disease stratification and prognosis. The specific patterns of circadian gene dysregulation in AML reveal a complex network of alterations that may contribute to disease pathogenesis and alter DNA damage repair mechanisms (Figure 2).

### 5.1. Clock Genes in AML

#### 5.1.1. BMAL1 and CLOCK

*Clock* and *Bmal1* play an important role in cellular proliferation and cell cycle progression in AML cells. In a recent study using a murine model of AML, it was shown that the genes *Bmal1* and *Clock* regulate the expression of leukaemic stem cells, with disruption of the circadian pathway producing anti-leukaemic effects such as impaired differentiation and depletion of leukaemia stem cells (LSCs) [22]. *BMAL1* expression was found to be significantly upregulated in bone marrow samples from human AML patients in comparison to healthy donors [57]. The level of *BMAL1* expression was found to correlate with cytogenetics with high *BMAL1* expression associated with poor survival.

Interestingly, in peripheral blood, the opposite was found. Human studies reported transcriptional silencing of the CpG island promoter of *BMAL1* in AML in 19% of studied patients [58]. Transcriptional analysis of the peripheral blood of newly diagnosed AML patients found that *BMAL1* expression was significantly down-regulated compared with healthy individuals, while *PER1*, *PER2*, *PER3*, *CRY1*, *CRY2*, *CLOCK*, and *TIM* were downregulated [59]. Another study showed that *BMAL1* was the most down-regulated gene among *CRY1*, *CRY2*, *CLOCK*, *PER2*, and *REV-ERBα* in PBMCs from AML patients [60]. The tissue-dependency of *BMAL1* expression levels, i.e., upregulated in bone marrow but downregulated or silenced in peripheral blood, is however not surprising, given other similar apparent expression discrepancies for AML indicator genes [61]. Although a definitive explanation has not been reached, potential mechanisms for the observed *BMAL1* tissue specificity may include niche-specific proliferation and differentiation signalling [62], differential epigenetic silencing [58], heterogeneity of cell populations [63] or differences in disease stage across sampled individuals [64].

#### 5.1.2. NPAS2

As a paralog of *CLOCK*, *NPAS2* forms heterodimers with *BMAL1* and compensates for *CLOCK* in regulating E-box–driven transcription [41]. *NPAS2* was found to be upregulated in AML patients, indicating that the knockdown in AML cells leads to cell cycle arrest at G1 and G2, and apoptosis [65].

#### 5.1.3. PERs and CRYs

PER1 and PER2 are widely considered tumour suppressors. In the peripheral blood of AML patients, the expression of *PER1*, *PER2*, *PER3*, *CRY1*, and *CRY2* has been found downregulated [59]. Increased blast cells in bone marrow correlated with decreased expression of *PER1* and *PER3*. Moreover, *PER1* and *PER3* expression were significantly upregulated in patients who achieved remission but remained low in those whose disease relapsed. *Per1* and *Cry1* were also suggested to be directly controlled by the *Clock* and *Bmal1* complex and to be mediators in *Clock* and *Bmal1* activity in AML [22]. Overexpression of *Per2* in haematopoietic cancer cell lines resulted in growth inhibition, cell cycle arrest, apoptosis, and loss of clonogenic abilities [66]. In acute promyelocytic leukaemia, caused by the fusion of the *PML* gene and the retinoic acid receptor alpha gene, it has been demonstrated that *PLM* physically interacts with *PER2* and that *PLM* is regulated by *PER2*; however, the specific mechanisms are still unclear [67].

#### 5.1.4. TIM

*TIM* facilitates the activation of ATR/CHK1 and ATM/CHK2 in the DDR [68]. In the peripheral blood of AML patients, *TIM* was shown to be downregulated compared with healthy individuals and upregulated in patients who achieved remission [59].

#### 5.1.5. CK1

*CK1ε* appears to be significantly upregulated in the peripheral blood of AML patients [59]. Functionally, *CK1ε* promotes proteasomal degradation of PER proteins; its upregulation in AML could accelerate PER turnover, facilitating cyclin accumulation and cell cycle progression [69,70].

#### 5.1.6. REV-ERBs and RORs

*REV-ERBα/β (NR1D1/2)* and *RORα/β/γ (RORA/B/C)* form an auxiliary circadian loop that fine-tunes *BMAL1* transcription: REV-ERBs repress BMAL1 by recruiting NCOR–HDAC3 co-repressor complexes, whereas RORs activate BMAL1 via RORE elements. In AML, *REV-ERBα* was found to be downregulated [60]. In contrast, *RORγ* was upregulated in a study of volunteers with AML [71]. This opens a new opportunity to investigate ROR levels in night-shift workers.

#### 5.1.7. Other Regulators

*SHARP1*, a suppressor of *CLOCK* and *BMAL1*, is upregulated in MLL-AF6 AML, while *REV-ERBα* and *PPARα*, which normally repress *BMAL1* or modulate metabolic clocks, are often reduced, suggesting altered circadian–metabolic crosstalk [72]. Co-repressors *NCOR1/2* are recruited by AML fusion proteins (e.g., AML1/ETO), enforcing chromatin compaction around differentiation genes [73].

### 5.2. Role of Circadian Genes in DNA Damage Response

**Figure 3:**
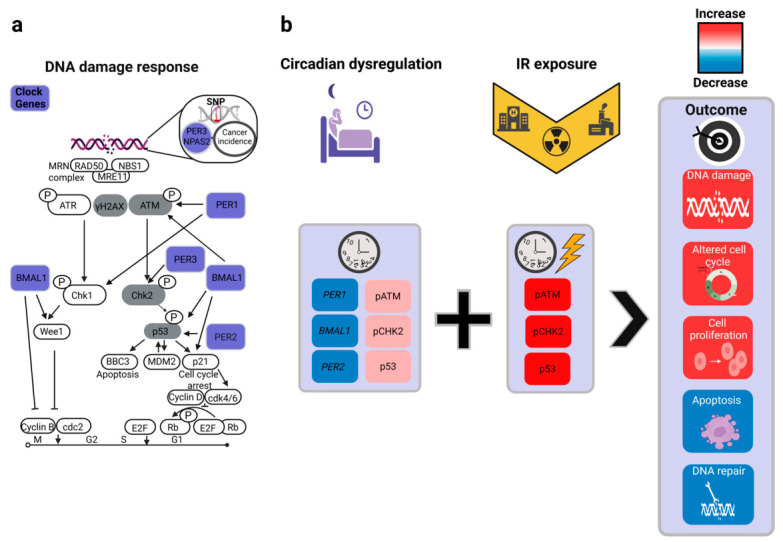
Mapping the influence of ionising radiation on core circadian clock genes and DNA-damage responses with potential relevance to radiation-induced acute myeloid leukaemia (rAML). (**a**) Role of the circadian genes in the DNA damage response and influence of PER3 and NPAS2 SNPs in HeLa and human non-Hodgkin’s lymphoma cell models on cancer incidence. *BMAL1* in mouse HSPCs and cardiac cells, human nasopharyngeal carcinoma cells, adrenocortical carcinoma cells and keratinocytes; *PER1* in rat glioma tissue; *PER2* in mouse thymocytes, embryonic fibroblasts and rat glioma tissue; *PER3* in human cervical carcinoma cells. (**b**) Colours indicate direction of reported change in *PER1*, *BMAL1*, *PER2* transcriptional changes and ATM, CHK2, P53 protein levels (red increase; blue decrease) after circadian dysregulation and circadian dysregulation combined with IR exposure in human peripheral blood. This leads to outcomes which could be explored as contributing to the development of rAML. Created in BioRender. O’Brien, G. (2026) https://BioRender.com/k7zxc4c accessed on 18 May 2026.

It has been established that CGs play a key role in cell cycle regulation. This regulation also extends to the DNA damage response pathway (Figure 3a), albeit current evidence predominantly emerges from non-haematopoietic systems, thus observations remain to be validated in cell populations relevant to AML development. Circadian disruption leads to desynchronized expression of CGs such as *PER1*, *PER2*, and *BMAL1* in human PBMCs [74]. Furthermore, simulated night-shift work results in increased endogenous and IR-induced levels of pATM, pCHEK2, and P53 compared to non-shift conditions [30], which altogether may lead to molecular and cellular outcomes favourable to the development of rAML (Figure 3b).

As previously discussed, BMAL1 suppresses p21 and regulates Wee1 and Cyclin B. Further roles of BMAL1 in the DDR have also been established with BMAL1 capable of directly binding to the p53 gene promoter region, activating the DDR pathway in a p53-dependent manner [75]. Also, a ChIP assay in mouse blood samples showed that Bmal1 protein binds directly to the Atm promoter region [76]. PER2 interacts directly with p53, protecting it from MDM2-mediated ubiquitination and degradation, thereby stabilising p53 activity and enhancing transcription of pro-arrest genes such as p21 [77]. BMAL1 further indirectly regulates this pathway through its repression of its downstream PER1/PER2 [78]. Per3 depleted HeLa cells resulted in impaired Chk2 activation, while overexpression of *Per3* led to increased phosphorylation of Chk2 and activation of the ATM-Chk2 pathway [79]. Polymorphisms of the genes *PER3* [80] and *NPAS2* have also been associated with cancer risk [81].

### 5.3. Clock Genes After Irradiation

#### 5.3.1. BMAL1 After Irradiation

Only a limited number of studies have investigated the expression of CGs after IR exposure, and none have examined the link to AML development to our knowledge. Nonetheless, *BMAL1* expression is positively correlated with increased radiosensitivity in nasopharyngeal carcinoma and keratinocytes [82]. After irradiation in the heart, Bmal1 protein levels were inversely associated with DNA damage levels, and Bmal1 depletion increased IR-induced DNA damage and apoptosis, suggesting that Bmal1 might protect against IR-induced toxicities [76]. In adrenocortical carcinoma cells, BMAL1 depletion significantly enhanced the sensitivity of cells to DNA damage-based therapies [83]. In contrast to these tumour cell models, one study showed that Bmal1−/− haematopoietic stem and progenitor cells exhibited unexpected radio-resistance, efficiently rescuing lethally irradiated wild-type hosts and sustaining long-term, multilineage reconstitution [84].

#### 5.3.2. NPAS2 Expression in DNA Damage Response

NPAS2 was found to enhance the stability of H2AX mRNA and to decrease tumour cell sensitivity by augmenting DDR. Evidence from breast cancer models and non-Hodgkin’s lymphoma shows that NPAS2 loss impairs DDR and reduces DNA repair capacity, underscoring its role in genomic stability [45]. Whether similar regulation occurs in AML remains to be determined.

#### 5.3.3. PERs and CRYs After Irradiation

In human cancer cells, *PER1* overexpression results in increased DNA damage-induced apoptosis [85]. On the other hand, one study showed that deletion of *Per2* promotes cancer following exposure to γ-irradiation in wild-type mice thymocytes suggesting that *Per2* functions in tumour suppression [21]. Consistently, *PER2* loss in mouse embryonic fibroblasts disrupts ATM–Chk2–p53 signalling, attenuating the DNA damage response and accelerating radiation-induced tumorigenesis. Restoration of *PER2* stabilises p53 and enhances apoptosis following genotoxic stress, underscoring its role as a key tumour-suppressive regulator of IR responsiveness [86]. In glioma tissue, high expression of *PER1* and *PER2* is associated with increased sensitivity to X-ray irradiation. Enhanced expression might improve the efficacy of radiotherapy against glioma by promoting apoptosis [87,88].

CRY1, upregulated by IR-induced DNA damage in prostate cancer cell lines, modulates the expression of homologous recombination repair genes, and is regarded as a pro-tumorigenic factor [89].

#### 5.3.4. REV-ERBs and RORs After Irradiation

Direct studies examining how REV-ERB or ROR signalling modulates irradiation responses in AML or normal haematopoietic cells remain limited. IR was found to reduce the mRNA expression of all three RORs in mouse testicular tissue and epididymis [90]. Preliminary data showed that RORα antagonist SR1001 and Rev-Erbα agonist GSK4112 significantly enhanced radiation-induced type I IFN activation [91].

## 6. Epigenetic and RNA-Based Modulators

Circadian regulation is not limited to transcriptional–translational feedback loops of core CGs but extends into broader epigenetic and post-transcriptional layers of gene regulation. Epigenetic modifiers and RNA-modifying enzymes cooperate with circadian proteins to fine-tune gene expression, chromatin accessibility, and state-dependent impact on IR-induced damage and DNA repair [92]. Here, we explore the additional concepts that might link AML, CGs, and IR in a broader epigenetic perspective.

### 6.1. m^6^A RNA Modification

Circadian clocks interact with RNA methylation pathways. One of the most prominent RNA modifications, N^6^-methyladenosine (m^6^A), dynamically regulates mRNA stability, splicing, and translation. The methyltransferase METTL3, in complex with METTL14, functions as a key writer of m^6^A marks, while demethylases such as FTO and ALKBH5 act as erasers. A third group of proteins, known as readers, regulate m^6^A metabolism by determining the downstream effects of m^6^A modifications such as YTHDF2, YTHFDF1, YTHDF3, YTHDC1, YTHDC2, IGF2BP1/2/3 and members of the HNRNP family. BMAL1 was shown to regulate rhythmic expression of m^6^A regulators, while methylation itself can confer temporal control on RNA stability and translation efficiency in hepatic lipid metabolism [93]. Ck1δ is regulated by m^6^A and when m^6^A is inhibited, the expression of Ck1δ isoforms increases. This increase slows the clock by increasing phosphorylation of PER2 at a key residue, stabilising the PER2 protein [94]. In AML, METTL3/METTL14 is consistently upregulated and promotes leukaemic cell self-renewal by enhancing translation of oncogenic transcripts, including c-MYC, BCL2, and PTEN [95]. FTO supports leukaemogenesis by demethylating pro-apoptotic transcripts, suppressing cell death and maintaining stemness. Pharmacological inhibition of FTO with FB23-2, reduces AML cell viability and enhances differentiation, demonstrating therapeutic potential [96]. ALKBH5 has been shown to be overexpressed in AML, and its increased expression correlates with poor prognosis in AML patients, particularly in t(8;21) AML [97].

In the context of radiation exposure, m^6^A levels are rapidly and transiently altered within minutes of exposure [98], with site-specific changes that may regulate the stability and turnover of stress-responsive transcripts during early radiation response. γ-Irradiation (4 Gy) in mouse bone marrow provokes rapid, reversible remodelling of the m^6^A epitranscriptome and transcriptome, with a characteristic 5 min increase and 2 h decrease in m^6^A fold-enrichment and mRNA abundance, and early enrichment of coding-sequence peaks [99,100].

### 6.2. Sirtuins and Chromatin Regulation

Sirtuins are NAD^+^-dependent histone deacetylases that integrate circadian metabolic cues with epigenetic programming. SIRT1 deacetylates both BMAL1 and PER2, shaping circadian oscillations of transcription [101]. In AML cells, SIRT1 and SIRT2 are frequently overexpressed, promoting leukaemic cell survival under stress by enhancing DNA repair and maintaining redox balance [102,103]. SIRT2 inhibition induces apoptosis in AML cells and sensitises them to chemotherapy, while dual inhibition of sirtuins can disrupt DDR, potentially enhancing radiation sensitivity [102].

## 7. Inflammation as a Link Between AML, DNA Damage Repair, and Circadian Rhythms

Inflammation is a hallmark of cancer promoting its progression [104]. Recent literature demonstrates a link between circadian disruption, inflammatory signalling, DNA damage responses and cancer biology. Thus, circadian misalignment induced by night-shift work, irregular light exposure or behavioural desynchrony alters transcriptional programmes that govern DNA repair, cell cycle progression and immune surveillance might contribute to AML development [105,106].

### 7.1. Clock Gene Expression, Inflammation, and Cancer

Studies suggest that cancer is associated with both immunostimulation and immunosuppression, mediated by increased levels of cytokines such as TNF-α and IL-6, and that systemic therapies, such as radiotherapy, may trigger additional inflammatory responses [106]. The regulation of the inflammatory response may be linked to disrupted CGs; in human glioblastoma, downregulation of BMAL1 and CLOCK induced glioblastoma stem cell cycle arrest and apoptosis, whereas upregulation of CLOCK promoted immune suppression [107]. In breast cancer, high CLOCK expression correlates with reduced CD8+ T-cell infiltration and increased M2 macrophage polarisation, consistent with increased PD-L1 expression [108].

### 7.2. Circadian Dysregulation, Inflammation, and Cancer

Clock disruption has also been shown to drive cancer progression by promoting the accumulation of myeloid-derived suppressor cells (MDSCs) and reducing the proportion of CD8+ cytotoxic T-cells [105]. MDSCs are present at very low numbers in healthy subjects but can expand significantly in malignant, infectious, and chronic inflammatory diseases, including AML, although few studies have examined MDSCs in acute leukaemias [109]. Similarly, simulation of chronic jet lag (advancing the light-dark cycle by 6 h every 2 days) has been suggested to induce widespread reprogramming of immune and metabolic pathways and to alter tissue-level glucose uptake, demonstrating that circadian disruption can reshape oncogenic signalling environments *in vivo* [110]. Long-term circadian misalignment accelerates immune senescence, increases *PD-1*-Positive CD44-high T-cell populations, and expands germinal centre B cells, indicating that persistent inflammatory activation arises directly from clock disruption and may create a permissive niche for malignant transformation in myeloid progenitors [111]. Night-shift work and artificial light at night produce internal desynchrony between central and peripheral oscillators and alter peripheral blood mononuclear cell CG expression, suggesting that the haematopoietic compartment is directly sensitive to environmental timing cues that are frequently disrupted in modern societies [112].

In this context, melatonin has emerged as a central player between circadian dysregulation, chronic inflammation, and cancer [113]. Melatonin is a circadian hormone with many well-established anticancer functions such as apoptosis, oxidation and inhibition of angiogenesis, metastasis, and energy production [114,115,116]. In tumour cell lines of human haematopoietic origin, melatonin was shown to exert a pro-oxidant effect by stimulating production of intracellular reactive oxygen species (ROS) which was associated with significant cytotoxicity and activation of caspases [117]. In mixed lineage leukaemia-rearranged (MLL-R) cell lines, melatonin was shown to inhibit cell proliferation and induce cell apoptosis through RBFOX3/hTERT and NF-KB/COX-2 signalling pathways, which have roles in progression of cancer and association with poor prognosis [118]. In FLT3/IDT AML cells, melatonin reduced proliferation and induced apoptosis through an increased release of cytochrome c from the mitochondria to the cytosol, vital for apoptosis [119]. Melatonin therefore has a varied yet integral role in leukaemia. In human patient samples, serum concentrations of melatonin were lower in CLL patients in comparison to healthy donors, and a lower level of melatonin was also reported in shift workers in comparison to non-shift workers, illustrating the strong effect shift work has on melatonin production [120].

### 7.3. DNA Damage Response, Inflammation, and Cancer

Ionising radiation initiates a tightly interconnected network of DDR and inflammatory signalling which could collectively shape cancer progression and therapeutic outcome. One of the links between DDR, inflammation, and cancer could be through the cGAS-STING activation pathway. Unrepaired damaged chromosomes after IR exposure can lead to micronuclei formation and release of nuclear DNA to the cytosol upon micronuclei rupture where it can be detected by cGAS [121]. This converts genotoxic stress into innate immune activation through STING–TBK1–IRF3 signalling, driving type I interferons and pro-inflammatory cytokines [122]. This inflammatory axis enhances dendritic cell priming and cytotoxic T-cell recruitment, providing a mechanistic basis for radiation-induced immunogenicity [123]. Persistent cytosolic DNA and sustained STING activation can promote senescence-associated secretory phenotypes enriched in IL-6, IL-8, CCL2, and related chemokines, fostering immunosuppression, fibrosis, and tumour-supportive remodelling. Macrophages exposed to radiation adopt a mixed inflammatory phenotype, secreting IL-6, IL-1β, TNF-α, and CCL chemokines that can either support anti-tumour immunity or drive pathological inflammation. High radiation doses also induce TREX1, degrading cytosolic DNA and suppressing cGAS activation, thereby limiting beneficial immune priming while permitting residual inflammatory damage [123]. The cGAS and STING expression has been found to be elevated in AML patients compared to healthy controls with a higher NRAS/KRAS mutation rate and lower complete remission rate [124]. Single nucleotide polymorphisms (SNPs) in the cGAS-STING signalling pathways were also associated with AML and related to AML susceptibility, chemotherapy response, and AML overall survival [125]. Interestingly, STING was found to play a vital role in the MDSCs differentiation [126], which, in combination with MDSCs caused by the clock gene disruption, could drive cancer progression.

**Figure 4:**
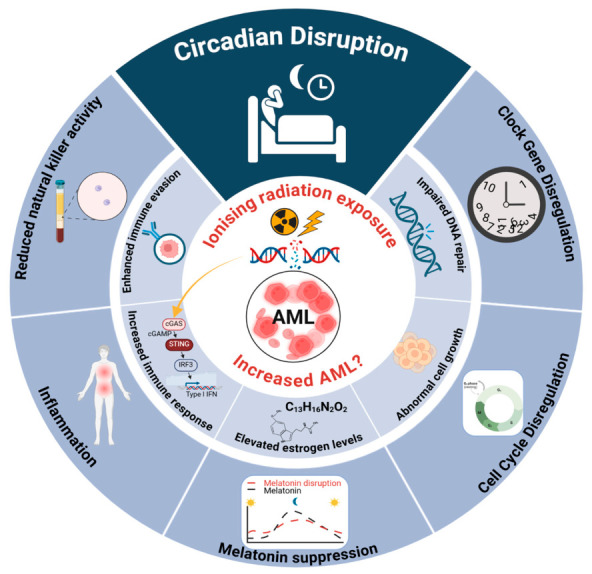
Proposed mechanistic model of how circadian rhythm disruption may contribute to rAML. Outer and intermediate blue circles: separate sources of experimental evidence (represented in segments) suggest that circadian disruption results in clock gene and cell cycle dysregulation, melatonin suppression, increased inflammation, and reduced natural killer activity, which may lead to impaired DNA repair, abnormal cell growth, elevated oestrogen levels, increased immune response, and enhanced immune evasion, as discussed in the text with references therein. Some of these effects, such as an increased activity of the cGAS-STING pathway, are also expected following ionising radiation exposure, a risk factor for AML. Inner white circle: Considering all the indirect evidence gathered in the text, we hypothesise that circadian disruption may interact with IR-induced responses towards an increased risk of AML development. This remains a gap in knowledge (white circle) to be validated experimentally. Created in BioRender. Lopez Riego, M. (2026) https://BioRender.com/kj532sf accessed on 18 May 2026.

These findings would suggest and support a model in which circadian disruption amplifies inflammatory signalling through cGAS-STING pathway after DDR, thereby creating conditions that may facilitate AML initiation and progression, opening avenues for further exploration (Figure 4).

## 8. Conclusions

Understanding how circadian disruption in the context of ionising radiation exposure may enhance AML development remains in the early stages. Evidence from basic research, epidemiology, and preclinical models has established that circadian clocks influence DNA repair, cell cycle control, and haematopoiesis. This review proposes a hypothetical mechanistic model describing the potential interaction and synergistic effects between circadian clock disruption and radiation-induced responses in AML pathogenesis (Figure 4) based on currently available evidence. Several areas stand out as priorities for future research. Firstly, defining how circadian phases alter repair fidelity, checkpoint activation, and apoptotic thresholds in normal and pre-leukaemic stem cells will clarify how circadian disruption contributes to leukaemogenesis, perhaps through inflammation. Secondly, incorporating circadian assessment into night-shift worker cohorts may uncover associations between circadian misalignment and AML incidence and/or latency. Prospective studies in radiotherapy patients that include monitoring of circadian biomarkers could provide the most substantial evidence. Then, core CGs and epigenetic regulators influenced by circadian timing hold potential as biomarkers for radiation sensitivity. Ultimately, in a broader context, these findings could have a significant impact on occupational and public health strategies. Policies addressing circadian health in radiation-exposed occupational groups, including healthcare workers and nuclear industry employees, may have a long-term preventive impact. If successful, these efforts could yield not only a deeper understanding of AML pathogenesis but also innovative strategies for policy prevention and chronoradiotherapy.

## Data Availability

No new data were created or analyzed in this study.

## Abbreviations

The following abbreviations are used in this manuscript:

AML	Acute myeloid leukaemia
CGs	Clock genes
CH	Clonal hematopoiesis
ChIP	Chromatin immunoprecipitation
CLL	Chronic Lymphocytic Leukaemia
DDR	DNA damage response
HSCs	Haemopoietic stem cells
IARC	International Agency for Research on Cancer
IL-6	Interleukin-6
IR	Ionising radiation
LSCs	Leukaemia stem cells
m^6^A	N^6^-methyladenosine
MDSCs	Myeloid-derived suppressor cells
MLL-R	Mixed lineage leukaemia-rearranged
NAD^+^	Nicotinamide adenine dinucleotide
NHL	Non-Hodgkin’s Lymphoma
NK	Natural Killer
PBMCs	Peripheral blood mononuclear cells
PCGs	Peripheral clock genes
PD-1	Programmed cell death protein 1
PD-L1	Programmed death-ligand 1
rAML	Radiation-induced acute myeloid leukaemia
SNP	Single nucleotide polymorphisms
TFS	Transcription factors
TNF-α	Tumour necrosis factor alpha
TTFLs	Transcriptional-translational feedback loops
